# Unveiling the dance of evolution: Pla-mediated cleavage of Ymt modulates the virulence dynamics of *Yersinia pestis*

**DOI:** 10.1128/mbio.01075-24

**Published:** 2024-07-03

**Authors:** Gengshan Wu, Yazhou Zhou, Shiyang Cao, Yarong Wu, Tong Wang, Yu Zhang, Xiaoyi Wang, Yajun Song, Ruifu Yang, Zongmin Du

**Affiliations:** 1 State Key Laboratory of Pathogen and Biosecurity, Beijing Institute of Microbiology and Epidemiology, Beijing, China; Northern Arizona University, Flagstaff, Arizona, USA

**Keywords:** Pla protease, *Yersinia *murine toxin, cleavage, virulence dynamics

## Abstract

*Yersinia pestis* has recently evolved into a highly lethal flea-borne pathogen through the pseudogenization of extensive genes and the acquisition of exogenous plasmids. Particularly noteworthy are the newly acquired pPCP1 and pMT1 plasmids, which encode the virulence determinants Pla and *Yersinia* murine toxin (Ymt), crucial for subcutaneous infection and survival within flea vector of *Y. pestis*, respectively. This study reveals that Pla can cleave Ymt at K299 both *in vivo* and *in vitro. Y. pestis* expressing Ymt_K299A_ displays enhanced *in vitro* biofilm formation and increased blood survival, indicating significant roles of Pla-mediated Ymt cleavage in these phenotypes. Intriguingly, although both the ancestral form of Pla and the prevalent Pla-I259T variant in modern *Y. pestis* strains are capable of cleaving Ymt at K299, the cleavage efficiency of Pla-I259T is only half that of the ancestral variant. In subcutaneous infection, mice infected with Δ*ymt::ymt-*K299A show significantly prolonged survival compared to those infected with Δ*ymt::ymt*. Similarly, infection with Δ*pla::pla-*I259T also results in extended survival compared to Δ*pla::pla* infection. These data demonstrate that the I259T substitution of Pla mitigates the enhanced virulence of *Y. pestis* in mice caused by Pla-mediated Ymt cleavage, thereby prolonging the survival period of infected animals and potentially conferring advantages on the transmission of *Y. pestis* to the next host. These findings deepen our understanding of the intricate interplay between two newly acquired plasmids and shed light on the positive selection of the Pla-I259T mutation, providing new insights into the virulence dynamics and transmission mechanisms of *Y. pestis*.

**IMPORTANCE:**

The emergence of *Y. pestis* as a highly lethal pathogen is driven by extensive gene pseudogenization and acquisition of exogenous plasmids pPCP1 and pMT1. However, the interplay between these two plasmids during evolution remains largely unexplored. Our study reveals intricate interactions between Ymt and Pla, two crucial virulence determinants encoded on these plasmids. Pla-mediated cleavage of Ymt significantly decreases *Y. pestis* survival in mouse blood and enhances its virulence in mice. The prevalent Pla-I259T variant in modern strains displays reduced Ymt cleavage, thereby extending the survival of infected animals and potentially increasing strain transmissibility. Our findings shed light on the nuanced evolution of *Y. pestis*, wherein reduced cleavage efficiency is a positive selection force, shaping the pathogen's natural trajectory.

## INTRODUCTION

*Yersinia pestis*, recognized as the causative agent of the notorious plague, stands as a testament to the dynamic nature of bacterial evolution. Its recent evolution from a closely related ancestor, *Yersinia pseudotuberculosis*, a food and water-borne enteric pathogen, into a flea-borne pathogen causing lethal systematic infection, marks a crucial chapter in the annals of infectious diseases (1, 2). This evolutionary journey hinges significantly on the acquisition of two exogenous plasmids, namely pPCP1 and pMT1, accompanied by an extensive pseudogenization of genes involved in gastric intestinal colonization (3). Remarkably, only four minor genetic changes, that is, acquisition of *ymt* and mutation of *rcsA*, *pde2,* and *pde3* are adequate for flea-borne transmission (4). This adaptability underscores the remarkable plasticity of *Y. pestis* in reshaping its pathogenic strategy. One of the key tactics employed by *Y. pestis* in its interaction with its vector, the flea, involves the formation of cohesive bacterial biofilms that obstruct the flea foregut. This process induces starvation and triggers intensified feeding behavior in fleas, ultimately leading to the regurgitative transmission of *Y. pestis* to new hosts (5). This highlights the intricacies of the host-pathogen-vector relationship and emphasizes the ecological consequences of *Y. pestis* evolution.

The colonization and persistence of *Y. pestis* within the flea midgut are intricately linked to the phospholipase D (PLD) activity of the *Yersinia* murine toxin (Ymt), a protein encoded on the newly acquired pMT1 plasmid (6). Ymt harbors two conserved motifs, HXKX_4_DX_6_G(G/S), with histidine (H188, H525) and lysine (K190, K527) playing a critical role in its catalytic activity (6, 7). Mutation of these critical amino acid residues results in a reduction of over 99% in the PLD activity of Ymt (7). This enzymatic activity is crucial for shielding *Y. pestis* against toxic metabolites generated during the digestion of red blood cells in fleas, with a more pronounced effect observed in blood meals from mice, humans, and black rats compared to brown rats (6, 8). *Y. pestis* deficient in PLD activity of Ymt has a greatly reduced ability to form cohesive aggregates, hindering the obstruction of the flea foregut and leading to elimination within 1 week (6). However, the correlation between the PLD activity of Ymt and the biofilm formation capability of *Y. pestis* remains unexplored.

The transmission of *Y. pestis via* fleas is a critical phase in its life cycle, and it closely associates with a high concentration of circulating bacteria in the bloodstream of hosts. Following the bite of an infected flea, bacteria enter the rodent’s body and are readily engulfed by local phagocytes. Subsequently, they travel along lymphatic vessels to the draining lymph nodes, where extensive bacterial replication occurs, leading to the onset of bubonic plague. As the disease progresses, *Y. pestis* disseminates into the blood vessels, resulting in a secondary septicemic plague characterized by overwhelming toxemia (1). Given that the blood meal volumes of individual fleas are exceptionally small (8, 9), the establishment of end-stage high-titer bacteremia in hosts becomes imperative for ensuring enough bacteria are ingested by fleas. The colonization of *Y. pestis* in fleas allows for the initiation of an effective infection, thereby sustaining the transmission cycle between fleas and hosts. However, factors influencing the survival of *Y. pestis* in the host blood remain unknown, presenting a critical knowledge gap in understanding the dynamics of *Y. pestis* pathogenesis.

The Pla protease, encoded on the pPCP1 plasmid unique to *Y. pestis*, emerges as a significant virulence determinant in the context of the bacterium’s interaction with its mammalian host. The acquisition of Pla alone is sufficient for the dissemination of bacteria from the initial subcutaneous infection site into the lymphatic system to cause bubonic plague (10, 11). In addition, Pla facilitates rapid replication of *Y. pestis* in the lungs, promoting the development of fulminant primary pneumonic plague in mammals (12). Pla belongs to the omptin family and exhibits remarkable ability to cleave various substrates *in vitro*, including plasminogen, α2-antiplasmin, and tissue factor pathway inhibitor. These substrates play crucial roles in the coagulation and fibrinolysis pathway (13). Despite the high conservation of the coding sequence of *pla* in ancestral *Y. pestis* lineages, a notable single amino acid substitution, I259T, in Pla emerged during evolution and is present in all modern lineages of *Y. pestis* strains (14). This intriguing mutation hints at the presence of a strong positive selection that confers advantages to *Y. pestis*.

Previous studies shed light on the unique characteristics of the Pla-I259T variant. This variant can self-process to generate the β-Pla isoform. In addition, it exhibits increased cleavage activity toward plasminogen (Plg) (14). Due to the critical role of Pla-mediated Plg activation in the dissemination of *Y. pestis* throughout the host body, the Pla-I259T mutation further augments bacterial invasiveness and contributes to the progression of bubonic plague. The impact of the Pla-I259T mutation, however, is likely contingent on the distinct physiological functions of various substrates of Pla. Notably, there might still be undiscovered substrates of Pla, the identification of which would not only enhance our understanding of this versatile virulence factor but also potentially unveil new impacts of the Pla-I259T mutation. This underlines the need for a comprehensive exploration of the interactions between Pla and its substrates to unravel the intricate mechanisms governing *Y. pestis* pathogenicity.

Here, we identify Ymt as a substrate of Pla, which is capable of cleaving Ymt into two fragments, both *in vitro* and *in vivo*. This cleavage, while not influencing the PLD activity of Ymt, impairs the *in vitro* biofilm formation of *Y. pestis*, hampers bacterial survival in mouse blood, and increases the virulence of *Y. pestis* in mice. The modern Pla variant, Pla-I259T, exhibits a reduced cleavage activity toward Ymt and contributes to the prolonged survival of infected hosts, which extends the time frame in which *Y. pestis* containing blood meals can be offered, thereby favoring their transmission. Thus, the Pla-I259T mutation might ultimately enhance the transmissibility of *Y. pestis*, serving as a positive selection force during the evolutionary trajectory of *Y. pestis*.

## RESULTS

### Pla protease is capable of cleaving Ymt at K299 both *in vivo* and *in vitro*

In our previous study, we incidentally detected a band with lower molecular weight in addition to the band of full-length Ymt. Intriguingly, this smaller band was only observed in culture supernatants of pPCP1^+^ strains but not in those from the pPCP1^−^ strains (data not shown). Considering pPCP1 encodes Pla that is a known protease showing activity on diverse substrates, we wonder whether Pla is potentially involved in this phenomenon.

**Fig 1:**
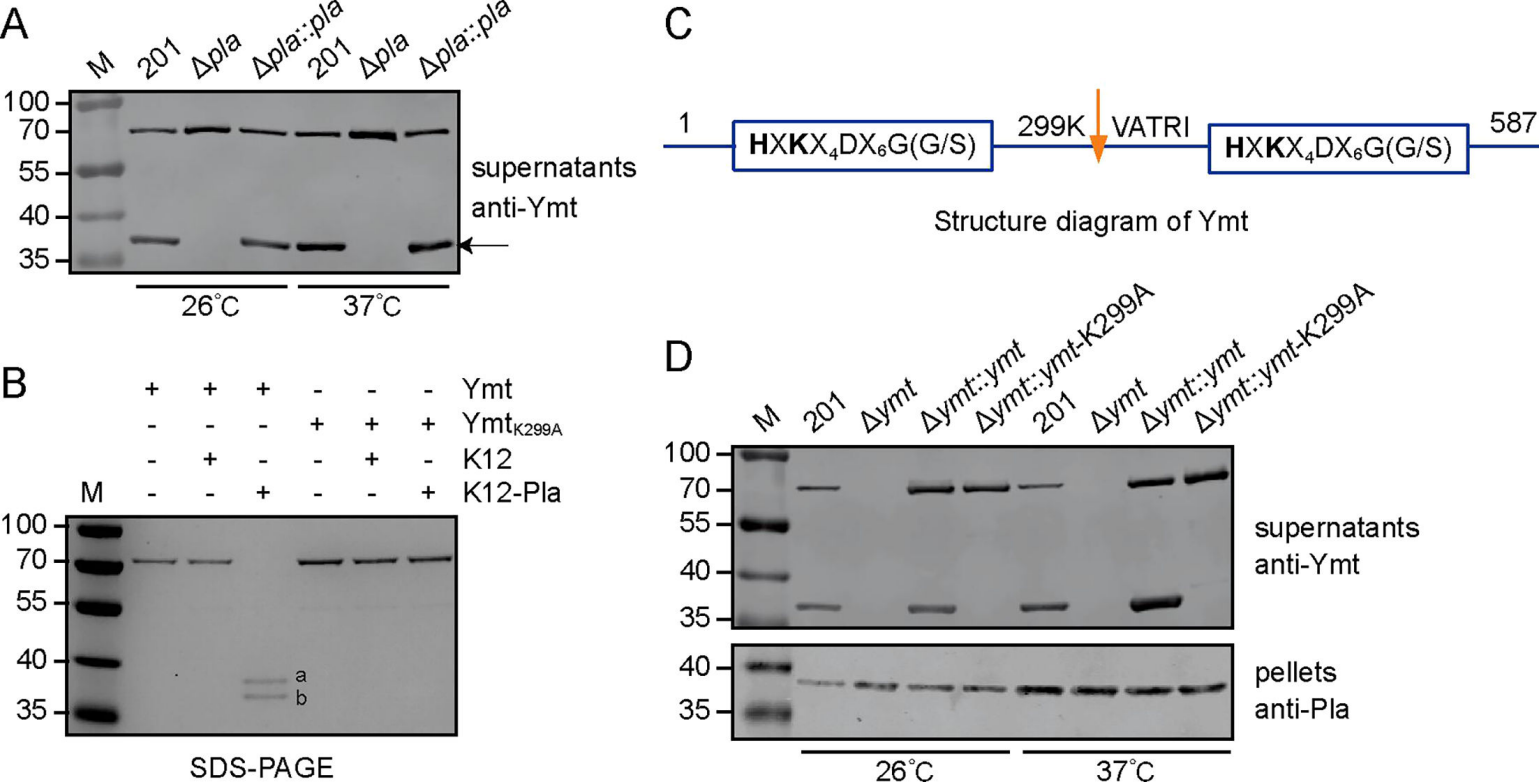
Pla is capable of cleaving Ymt both *in vivo* and *in vitro*. (**A**) *Y. pestis* strains 201, Δ*pla,* and Δ*pla::pla* were cultured in LB medium at 26°C or 37°C and the culture supernatants were collected by centrifugation. Proteins in the supernatants were TCA-precipitated and separated by 12% SDS-PAGE, followed by Western blotting using an anti-Ymt polyclonal antibody. Arrowhead indicated the cleavage product of Ymt. (**B**) The purified Ymt and Ymt_K299A_ were incubated with *E. coli* K12 containing a Pla-expressing plasmid or an empty vector for 2 h at 37°C. The products were analyzed as described above, with bands a and b indicating the two cleaved fragments of Ymt. (**C**) The Ymt fragments shown in (**B**) were cropped and subjected to N-terminal sequencing to identify cleavage sites within Ymt. (**D**) *Y. pestis* strains 201, Δ*ymt*, Δ*ymt::ymt*, and Δ*ymt::ymt*-K299A were cultured and processed as described in (**A**). Proteins in the supernatants were detected by Western blotting using anti-Ymt antibody, and proteins in the bacterial cell pellets were detected with an anti-Pla antibody.

To test this hypothesis, we constructed a *pla* deletion mutant (Δ*pla*) using *Y. pestis* strain 201 as the parent strain. Subsequently, the mutant was complemented with the Pla-expressing plasmid pACYC184, resulting in Δ*pla::pla*. Western blotting was employed to detect Ymt in culture supernatants of strains 201, Δ*pla*, and Δ*pla::pla* grown at 26°C or 37°C. A cleaved product of Ymt was observed in *Y. pestis* strain 201 and Δ*pla::pla*, but not in Δ*pla* (Fig. 1A), confirming our hypothesis that Pla is involved in the cleavage of Ymt. Moreover, the significant presence of Ymt in culture supernatants implies its extracellular secretion. To further assess Pla’s cleavage activity toward Ymt, the purified recombinant Ymt was incubated with *E. coli* K12 cells carrying either a Pla-expressing plasmid or an empty vector for 2 h. Two distinct cleaved fragments of Ymt were clearly detected when Ymt was incubated with K12-expressing Pla, whereas only a single band of the full-length Ymt was detected when incubated with K12 (Fig. 1B). These results indicated that Pla exhibits protease activity to Ymt, likely with a single cleavage site.

To identify the specific cleavage site, two Ymt fragments corresponding to bands a and b in Fig. 1B were collected and subjected to N-terminal sequencing analysis (Fig. S1). The results revealed a cleavage site between amino acids K299 and V300 of Ymt (Fig. 1C). Then, we generated a K299A variant of Ymt, termed Ymt_K299A_, to conduct the same assay as described above. It was found that Ymt_K299A_ remained intact after incubation with K12-expressing Pla for 2 h (Fig. 1B), confirming that Pla cleaves Ymt at K299A as revealed by N-terminal sequencing analysis.

To verify the cleavage site in Ymt *in vivo*, a *ymt* deletion mutant (Δ*ymt*) and complementary strains expressing Ymt or Ymt_K299A_ were constructed (Table S1). Immunoblot analysis showed detectable cleavage products of Ymt in culture supernatants of *Y. pestis* strains 201 and Δ*ymt::ymt*, but not in those from Δ*ymt::ymt*-K299A (Fig. 1D). These results indicated that Pla is capable of cleaving Ymt *in vivo* between amino acids K299 and V300. Furthermore, the proportion of cleaved Ymt in the culture supernatants of 201 and Δ*ymt*::ymt appears to be more abundant when bacteria were grown at 37°C compared to 26°C, consistent with previous findings that Pla expression is significantly upregulated at 37°C (Fig. 1D) (15, 16).

We noticed that *in vivo* assays revealed only one cleavage band of Ymt (Fig. 1A and D), while purified Ymt incubated with Pla-expressing K12 produced two distinct bands (Fig. 1B). We speculated that one of the cleaved Ymt peptides might be further degraded by some unknown mechanism in complex physiological conditions. In summary, these findings demonstrated that Pla directly cleaves Ymt into two fragments, both *in vivo* and *in vitro*, at K299.

### Pla-I259T exhibits reduced cleavage activity toward Ymt

**Fig 2:**
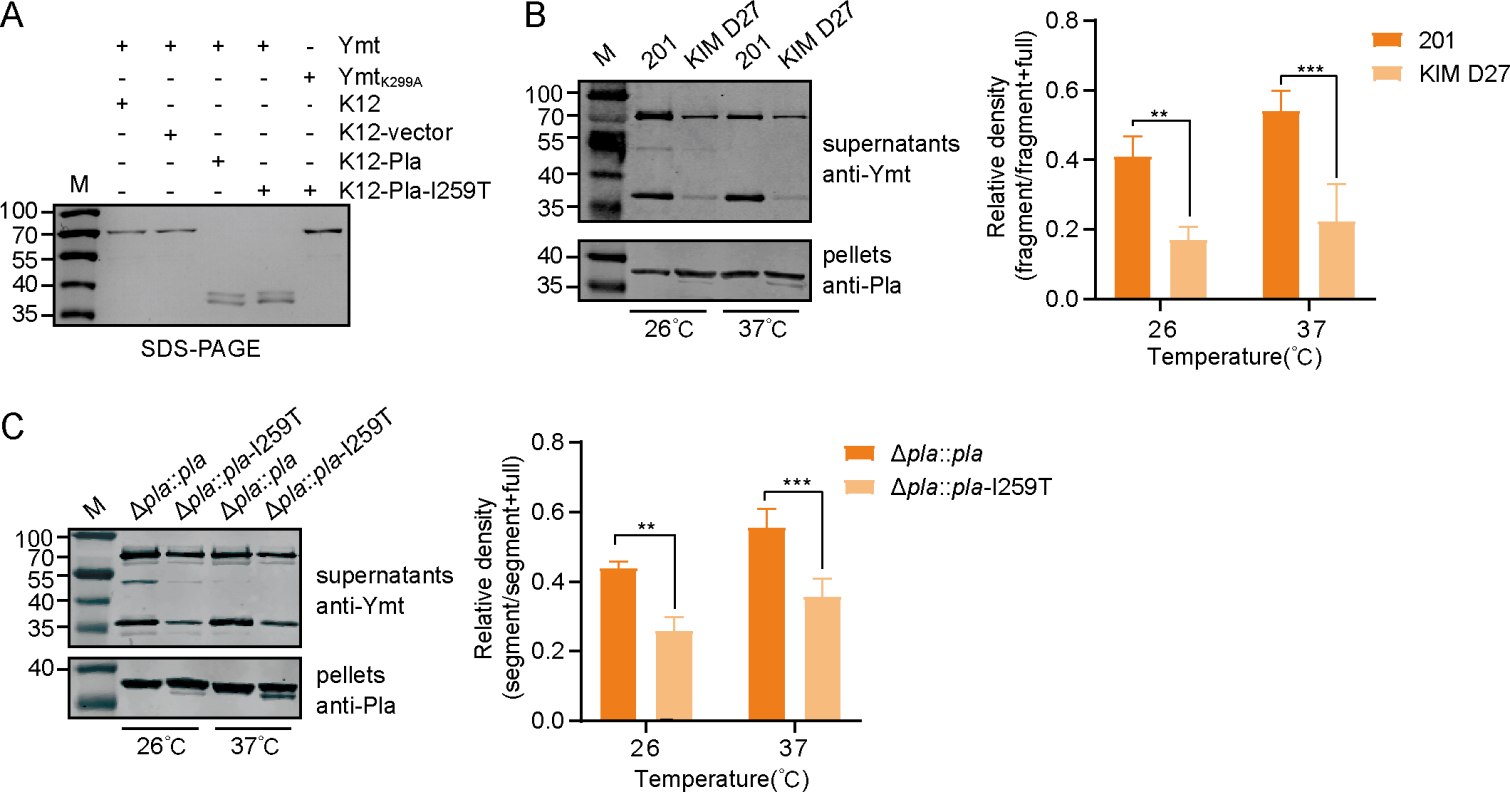
Pla-I259T cleaves Ymt between amino acids K299 and V300 as Pla does, albeit with a reduced efficiency. (**A**) The purified recombinant Ymt and Ymt_K299A_ were incubated with K12-expressing Pla or Pla-I259T for 2 h, followed by 12% SDS-PAGE separation. (**B**) *Y. pestis* strains 201 and KIM D27 were cultured at 26°C or 37°C. Culture supernatants were collected for Western blotting with anti-Ymt antibody and bacterial cells were detected with anti-Pla antibody. ImageJ (version 1.43u) was used to determine relative density. Bars represent mean ± standard deviation (SD) from three independent experiments. Two-way ANOVA with Tukey’s multiple-comparison test assessed significance (*P＜0.05; **P＜0.01). (**C**) Similar assays in (**B**) were performed with *Y. pestis* strain Δ*pla::pla* and Δ*pla::pla*-I259T.

The observed Pla-mediated Ymt cleavage described above was identified in *Y. pestis* 201, a strain belonging to the ancestral *Y. pestis* lineage. It has been well known that throughout the evolutionary process, all modern *Y. pestis* strains carry a single amino acid substitution of I259T in Pla. Notably, Pla-I259T has been shown to enhance its protease activity for Plg (12). Thus, we next aimed to investigate whether this substitution alters its activity toward Ymt. *In vitro* assays revealed that K12-expressing Pla-I259T cleaved Ymt into two distinct fragments but showed no activity toward Ymt_K299A_ (Fig. 2A), indicating cleavage activity of Pla-I259T toward Ymt between amino acids K299 and V300, akin to Pla.

*In vivo* assays also supported the cleavage activity of Pla-I259T toward Ymt. The Ymt cleavage fragments were successfully detected in the culture supernatants of *Y. pestis* KIM D27, a strain belonging to the modern *Y. pestis* lineage expressing Pla-I259T (Fig. 2B, left panel). Notably, KIM D27 exhibited lower cleavage activity against Ymt compared to strain 201 (Fig. 2B, right panel), despite Pla abundances being similar in the two strains. To further investigate whether this minor discrepancy in cleaving efficiency on Ymt was caused by the I259T mutation of Pla, rather than by the intrinsic distinct nature of 201 and KIM D27 strains, we construct Δ*pla::pla* and Δ*pla::pla-*I259T of 201 to analyze the full-length Ymt as well as the cleaved Ymt in the supernatant of the bacterial cultures. The results demonstrate a similar finding that Pla-I259T cleaved Ymt at a slightly lower efficiency in comparison to Pla even in an isogeneic background (Fig. 2C).

Structural modeling revealed significant changes in the binding interface between Pla and Ymt after the mutation of the 259th amino acid of Pla from Isoleucine (I) to Threonine (T) (Fig. S2). Despite the stability of both structural systems (Fig. S3A through F), the root mean square deviation (RMSD) value of Ymt-Pla-I259T (0.3578) was lower than that of Ymt-Pla (0.7291) (Fig. S3G and H), indicating that the structural alterations in Ymt-Pla-I259T were less pronounced, leading to a more stable interaction. These findings suggest that the I259T mutation in Pla may modulate the protein dynamics near its 259th amino acid, resulting in a tighter binding with Ymt, thereby influencing the cleavage efficiency.

Taken together, these data suggested that Pla-I259T cleaves Ymt at a reduced rate in comparison to Pla. This result well explains the observations that cleavage proportion of Ymt were lower in KIM D27 and Δ*pla::pla*-I259T than those in 201 and Δ*pla::pla*.

### *In vitro* cleavage of Ymt by Pla has no impact on its PLD activity and toxicity to mice

**Fig 3:**
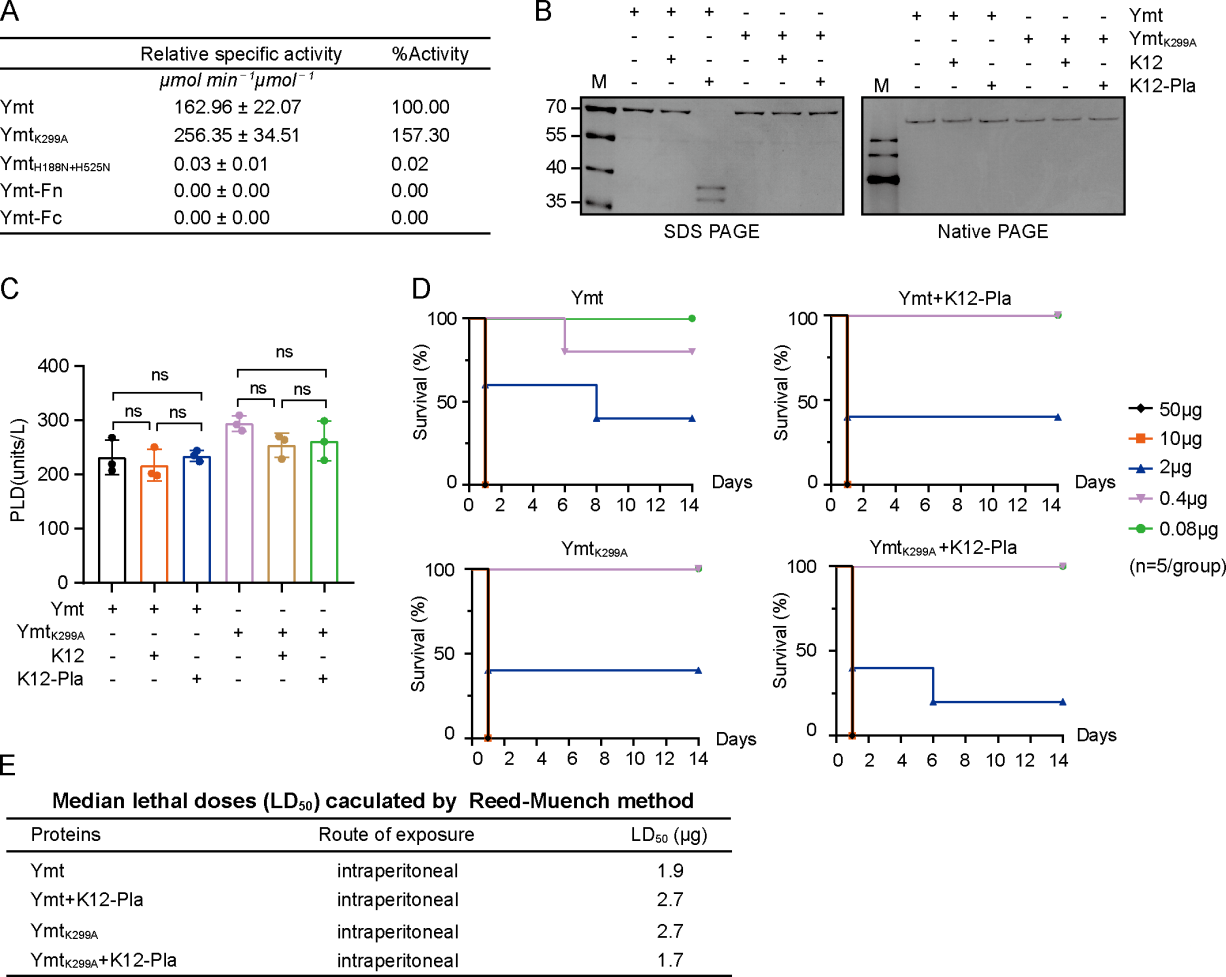
*In vitro* assessment of Pla-mediated cleavage impact on Ymt’s PLD activity and toxicity in mice. (**A**) Purified proteins (Ymt, Ymt_K299A_, Ymt_H188N+H252N_, Ymt-Fn, and Ymt-Fc) at equimolar concentrations were subjected to PLD activity measurement using a Phospholipase D activity assay kit, with Ymt as the standard. The relative specific activity refers to 1 µmol of indicated protein catalyzes the formation of 1 µmol choline per minute under the assay conditions. (**B**) Ymt and Ymt_K299A_ at matched concentrations were incubated with K12 or K12 containing Pla-expressing plasmid for 2 h. After removing bacterial cells, proteins in the supernatants were mixed with SDS or native sample loading buffer and analyzed by SDS-PAGE (left panel) or Native PAGE (right panel), respectively. (**C**) Proteins from (**B**) were subjected to PLD activity measurement as described in (**A**). Significance was determined using one-way ANOVA followed by Tukey’s multiple-comparison test. (**D**) Both Ymt and Ymt_K299A_, before and after their incubation with Pla-expressing K12 were subjected to fivefold serial dilutions, respectively. Five groups (*n* = 5) of female BALB/c mice (6 to 8 weeks old) were intraperitoneally challenged with proteins at varying dilutions and observed continuously for 2 weeks. Survival curves were analyzed using GraphPad Prism 8.0.1 software. (**E**) LD_50_ value for Ymt and Ymt_K299A_, pre- and post-incubation with Pla-expressing K12 in BALB/c mice *via* intraperitoneal route, were calculated using the Reed-Muench method.

As the PLD activity of Ymt is vital for *Y. pestis* survival in the flea midgut, we wondered whether cleavage by Pla influences its PLD activity. First, we constructed expression plasmids for Ymt, Ymt_K299A_, two Ymt truncations (Ymt-Fn and Ymt-Fc, the cleavage products of Ymt divided between K299 and V300), and Ymt_H188N+H252N_, a catalytic dead variant of Ymt with crucial histidine residues in PLD motif mutated to asparagine. The corresponding proteins were expressed in *E. coli* and purified by affinity purification. *In vitro* PLD activity measurements of those proteins revealed a slightly higher activity for Ymt_K299A_ than Ymt (Fig. 3A). Considering potential variations in measurements of protein purity and concentration, we concluded that K299A substitution in Ymt has no discernible effect on its PLD activity. In addition, we also observed that mutations in critical amino acid residues within conserved PLD motifs led to an almost complete loss of PLD activity. Interestingly, both Ymt truncations, despite reserving one of the intact conserved PLD motifs, showed no detectable PLD activity even at very high concentrations, which were consistent with the previous reports (7).

Next, to assess the impact of Pla-mediated cleavage on Ymt’s PLD activity, the purified Ymt and Ymt_K299A_ were individually incubated with *E. coli* K12 containing either Pla-expressing plasmid or an empty vector, as described above. The proteins were then collected by centrifugation and subjected to PLD activity measurement. Surprisingly, despite the clear observation of two fragments of Ymt produced by Pla cleavage in SDS-PAGE (Fig. 3B, left panel), the PLD activity of Ymt remained unaffected after incubation with Pla-expressing *E. coli* K12 (Fig. 3C). Further analysis using native PAGE electrophoresis revealed that cleaved Ymt products mediated by Pla exhibited a single band with a molecular weight equivalent to full-length Ymt (Fig. 3B, right panel). These results suggested that the two cleaved fragments of Ymt remained bound with each other after Pla-mediated cleavage, thereby preserving the PLD activity. Prediction of the tertiary structure of the Ymt-Fn and Ymt-Fc complex revealed a structure closely resembling the conformation of the intact Ymt monomer, suggesting that the cleavage does not significantly alter its molecular structure (Fig. S4).

Ymt has demonstrated a half lethality dose (LD_50_) of approximately 1 µg in mice, and the toxicity was attributed to its PLD activity (17). To assess the impact of cleavage by Pla on Ymt’s PLD activity *in vivo*, Ymt and Ymt_K299A_ were individually incubated with K12 containing Pla-expressing plasmid or the empty vector as described above. The recovered product proteins and the untreated Ymt and Ymt_K299A_ proteins were serially diluted and used to intraperitoneally challenge mice. The LD_50_ values of different proteins were determined using the Reed-Muench method. Results revealed that the toxicity of Ymt remained largely unaffected after Pla cleavage, with an LD_50_ close to the previously reported value of 1 µg (17). In addition, Ymt_K299A_ exhibited comparable toxicity to mice as Ymt (Fig. 3D and E). These findings showed that *in vitro* cleavage on Ymt by Pla has no discernible impact on its PLD activity as well as the toxicity in mice, probably due to that two cleaved fragments of Ymt may remain bound together to function as an active PLD.

### Cleavage of Ymt by Pla diminishes *in vitro* biofilm formation capabilities of *Y. pestis*

**Fig 4:**
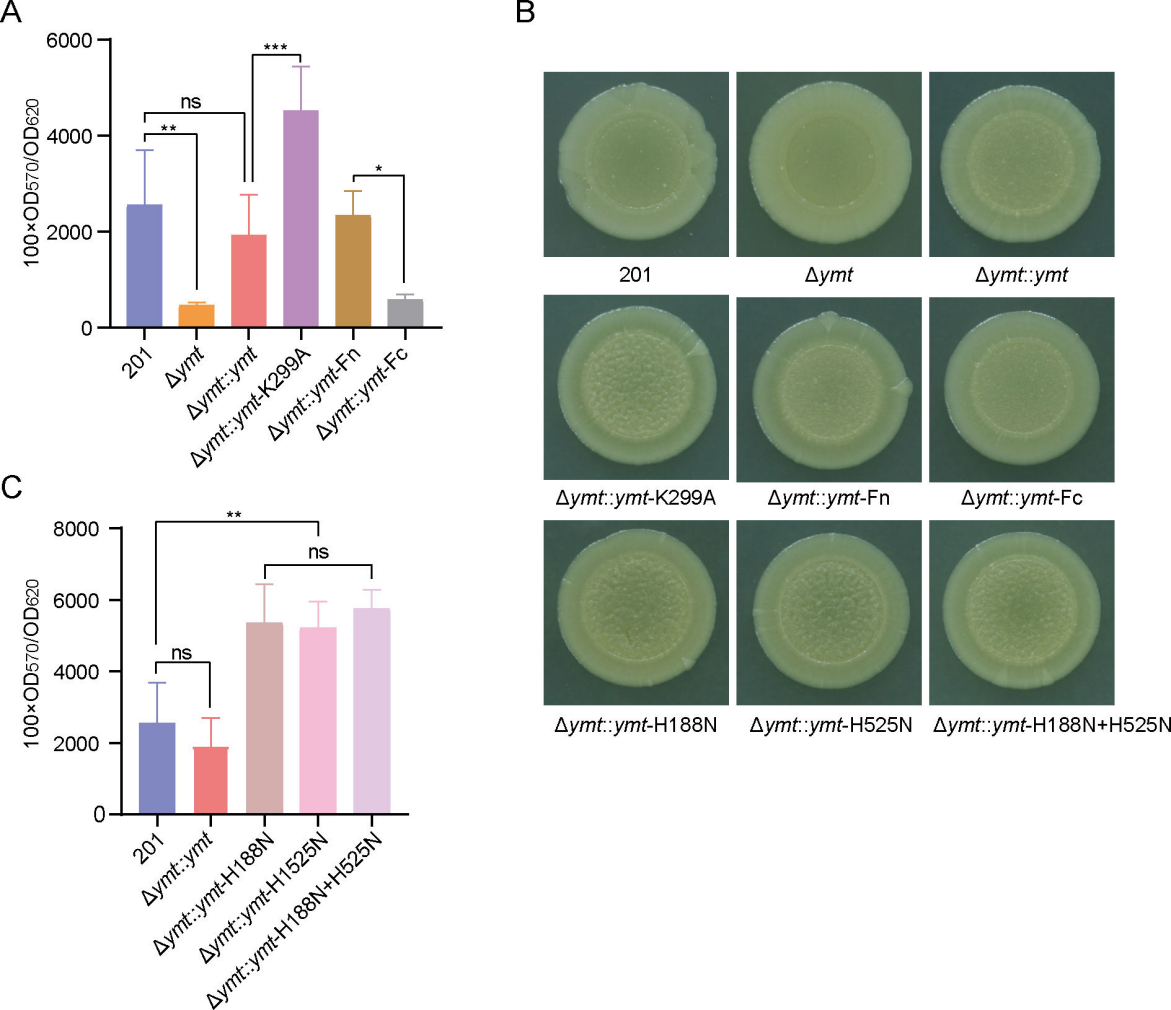
Ymt variants elicit divergent biofilm phenotypes in *Y. pestis* (**A and C**) *Y. pestis* strains were cultured in 24-well tissue culture plates at 26°C in LB medium. Planktonic cells were assessed for OD_620_ values, while crystal violet staining quantified bacterial biomass adhering to well walls by determining OD_570_ values. The relative capacity of biofilm formation capacity was calculated as 100 × OD_570_/OD_620_. Data represent mean ± SD from four biological replicates per strain. Significance was determined using one-way ANOVA followed by Tukey’s multiple-comparison test (*P＜0.05; **P＜0.01; and ***P＜0.001). Data are representative of three independent experiments. (**B**) Glycerol stocks of various *Y. pestis* strains were spotted on LB agar plates and incubated at 26°C for approximately 9 days. Representative images of typical colonies, captured at uniform magnifications, from at least three independent experiments.

The acquisition of *ymt* is considered advantageous for the formation of bacteria mass in the proventriculus, enabling flea-borne transmission of *Y. pestis* (18). To assess Ymt’s contribution to *Y. pestis* biofilm formation, crystal violet staining assays were performed. Bacterial strains were inoculated into 24-well tissue culture plates, and biofilms adhered on well walls were stained with crystal violet. Δ*ymt* exhibited significantly reduced biofilm formation compared to the wild-type strain, while Δ*ymt* complemented with a Ymt-expressing plasmid restored biofilm production to wild-type levels (Fig. 4A). These findings highlighted Ymt’s role in *Y. pestis* biofilm formation. Importantly, Δ*ymt::ymt*-K299A showed increased crystal violet staining compared to Δ*ymt::ymt*, suggesting that Ymt cleavage impairs *Y. pestis* biofilm formation (Fig. 4A). Notably, Δ*ymt* complemented with Ymt-Fn exhibited enhanced biofilm formation compared to Ymt-Fc complementation (Fig. 4A), suggesting a major role of Ymt-Fn in biofilm enhancing effect of Ymt. Biofilm formation by those strains was further confirmed through bacterial colony morphology assays, in which biofilm-producing capability was correlated with the roughness of colonies formed on LB agar plates (19). Consistently, we found that strains with robust biofilm formation in crystal violet staining assays exhibited a relatively rough colony morphology, and vice versa (Fig. 4B).

We further sought to examine the contribution of Ymt’s PLD activity to the biofilm formation of *Y. pestis*. Mutations of conserved histidine within PLD motifs impair the PLD activity (7). Thus, we generated complemented strains by introducing plasmids expressing specific Ymt mutants into Δ*ymt*, resulting in Δ*ymt::ymt*-H188N, Δ*ymt::ymt*-H525N, and Δ*ymt::ymt*-H188*N* + H525N. Colony morphology assays revealed that these mutants exhibited a more pronounced roughness than strains 201 and Δ*ymt::ymt* (Fig. 4B). Consistently, crystal violet staining demonstrated a significant increase in biofilm formation for strains expressing PLD-deficient Ymt variants (Fig. 4C). Considering that Ymt plays a crucial role in *Y. pestis* biofilm formation, we deemed that this enhancement could be attributed to the overexpression of Ymt in the complemented strains.

In summary, these results indicated that Pla cleavage of Ymt impairs the *in vitro* biofilm formation of *Y. pestis*, and this impairment has no relation to Ymt’s PLD activity.

### Ymt enhances *Y. pestis* survival in mouse blood, yet it is mitigated by Pla-mediated cleavage

**Fig 5:**
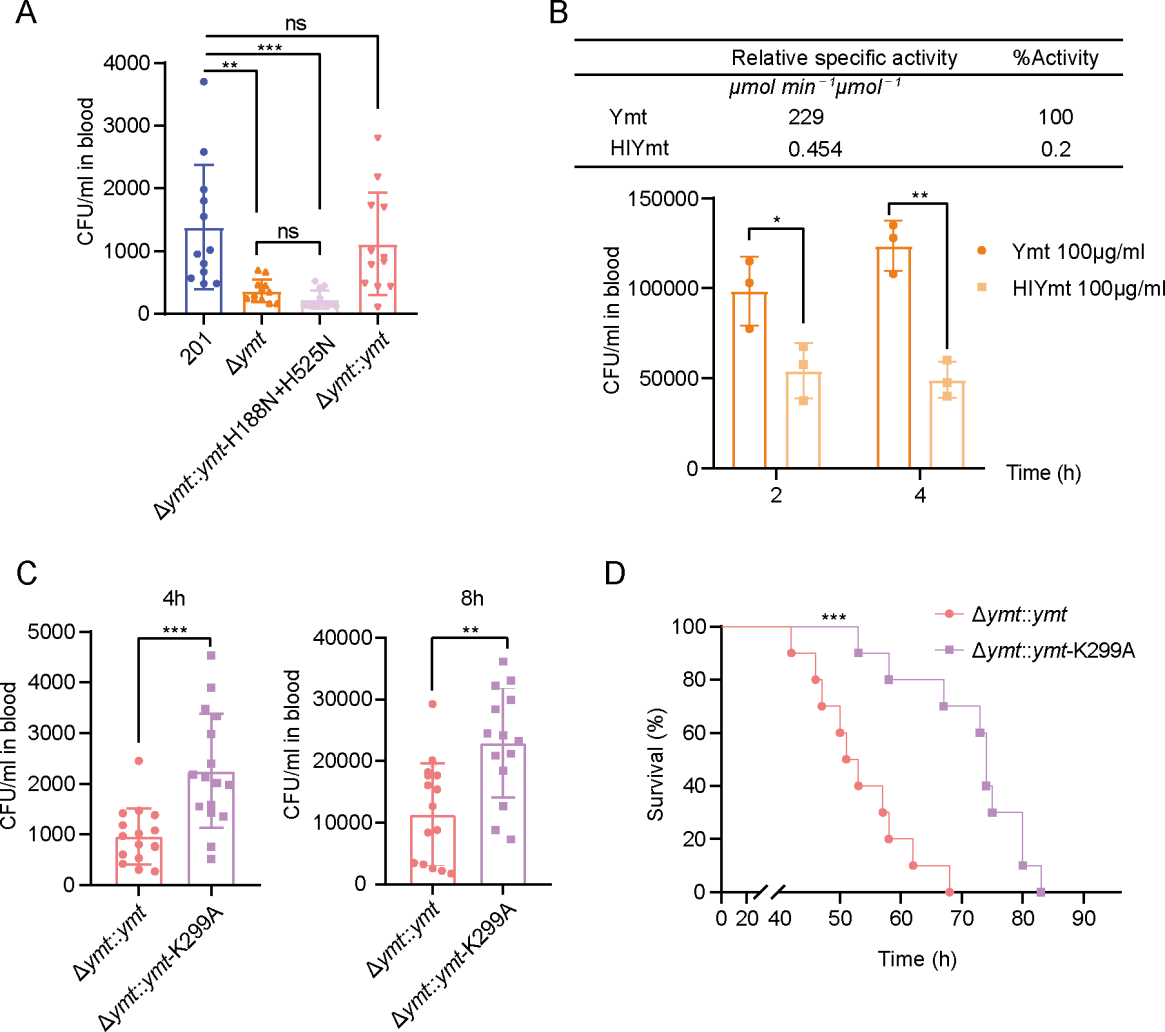
Differential impacts of Ymt variants on *Y. pestis* survival in mouse blood. (**A**) Groups of mice (*n* = 12) were *i.v*. injected with 10^6^ CFU of *Y. pestis* strains 201, Δ*ymt*, Δ*ymt::ymt*-H188*N* + H525N, or Δ*ymt::ymt*. After 4 h, mice were sacrificed, and blood samples were collected to determine bacterial counts. One-way ANOVA followed by Tukey’s multiple-comparison test was used to measure significance (**, *P*＜0.01; ***, *P*＜0.001). Data were collected from three independent experiments. (**B**) Fresh blood samples were collected from the retro-orbital plexus into tubes containing EDTAK2. The *Y. pestis* strain Δ*ymt::ymt*-H188*N* + H525N was inoculated into mouse blood supplemented with aliquots of Ymt or HIYmt (100 µg/mL) prior to incubation at 37°C. After 2 or 4 h, the number of live bacteria was assessed by plating serial dilutions of samples on agar plates. Significance was evaluated using a two-tailed unpaired Student’s *t*-test (*P＜0.05; **P＜0.01). Data represent one from two independent experiments, with each experiment conducted in triplicate. The PLD activity of HIYmt was measured as described above. (**C**) Groups of mice (*n* = 14) were challenged with *Y. pestis* strains Δ*ymt::ymt* and Δ*ymt::ymt*-K299A as described in (**A**). A two-tailed unpaired Student’s *t*-test was used to measure significance (**P＜0.01; ***P＜0.001). Data were collected from two independent experiments. (**D**) Groups of mice (*n* = 10) were challenged with *Y. pestis* strains Δ*ymt::ymt* or Δ*ymt::ymt*-K299A as described in (**C**). The survival time was recorded in hours, and statistical significance in the survival curves was analyzed using the Log-rank test in GraphPad Prism 8.0.1 (***P＜0.001).

Given that *Y. pestis* inhabits in blood-rich environment during its transmission process, either in flea digestive tracts or hosts with septicemic plague, its survival in host blood may intricately relate to the transmission efficiency. Therefore, we next evaluated the survival of various *Y. pestis* strains in blood post-infection using a mouse model simulating the terminal phase of septicemic plague. Mice were intravenously (*i.v*.) challenged with approximately 10^6^ CFU of various strains, and orbital blood samples were collected at 4 h post-infection (4 hpi.) and assessed for live bacteria counting. Notably, the number of Δ*ymt* bacteria was significantly lower than that of the wild-type strain (Fig. 5A), indicating a pivotal role of Ymt in promoting the survival of *Y. pestis* in blood. In addition, Δ*ymt* complemented with Ymt, but not Ymt_H188N+H252N_, restored the phenotype to wild-type levels (Fig. 5A), suggesting that this promotion effect depends on Ymt’s PLD activity.

The role of PLD activity of Ymt on *Y. pestis* survival in mouse blood was further examined using an *in vitro* assay. *Y. pestis* strain Δ*ymt::ymt*-H188*N* + H525N completely lacking PLD activity was inoculated into the mouse blood with the addition of Ymt or heat-inactivated Ymt (HIYmt), followed by incubation at 37°C. The inactivation of PLD activity of HIYmt was confirmed by phospholipase D assays, and it exhibits <1% PLD activity of Ymt protein (Fig. 5B, upper panel). The bacterial count in the blood samples of the Ymt-supplemented group significantly exceeded that of the HIYmt-supplemented group, and this effect was more pronounced at 4 hpi than 2 hpi (Fig. 5B, bottom panel). These results further confirmed that Ymt enhances *Y. pestis* survival in blood in a PLD activity-dependent manner.

To assess the impact of Pla-mediated cleavage of Ymt on *Y. pestis* survival in mouse blood, 10^6^ CFU of *Y. pestis* strains Δ*ymt::ymt* and Δ*ymt::ymt*-K299A were *i.v*. administered to mice to mimic the terminal phase of septicemic plague. At 4 hpi, Δ*ymt::ymt*-infected mice showed a significantly lower bacterial load in the blood (Fig. 5C, left panel) and milder symptoms compared to Δ*ymt::ymt-*K299A-infected mice, such as lethargy and ruffled fur. Similar difference in bacterial load between the two groups was observed at 8 hpi (Fig. 5C, right panel). However, long-term observation revealed that Δ*ymt::ymt*-infected mice were more susceptible to mortality, with a significantly shorter average survival time than mice infected with Δ*ymt::ymt-*K299A (Fig. 5D). These results suggested that although Pla-mediated cleavage of Ymt negates its enhancing effect on bacterial survival in the blood, it leads to a shortened survival time of infected mice in a model of severe septicemic plague. This observation suggests an unidentified mechanism linked to Ymt, influencing the virulence of *Y. pestis,* which needs further exploration.

Taken together, these findings indicated that Ymt’s PLD activity promotes *Y. pestis* survival in mouse blood, both *in vivo* and *in vitro*. Pla-mediated cleavage of Ymt attenuates the enhancing effect of Ymt on *Y. pestis* survival in mouse blood.

### The I259T mutation mitigates the enhanced virulence of *Y. pestis* in mice caused by Pla-mediated Ymt cleavage

**Fig 6:**
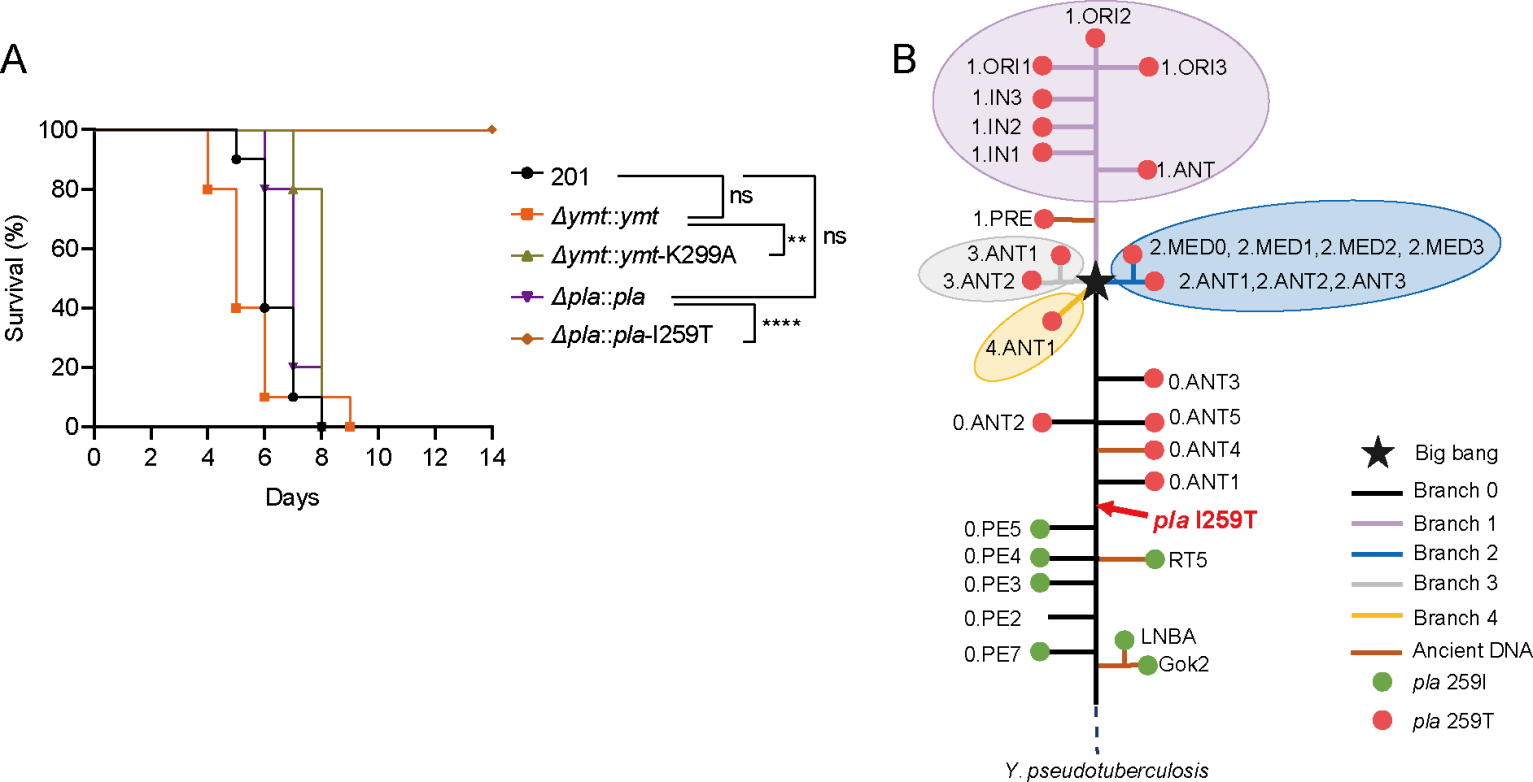
Two Pla variants exhibit distinct impacts on the virulence of *Y. pestis* in mice and belong to different evolutionary lineages. (**A**) Groups (*n* = 10) of BALB/c mice were *s.c*. challenged with 10 CFU of *Y. pestis* strain 201, Δ*ymt::ymt*, Δ*ymt::ymt*-K299A, Δ*pla::pla,* or Δ*pla::pla*-I259T. Mice received intraperitoneal injections of appropriate antibiotics twice daily to maintain plasmids in the complemented strains. All mice were observed continuously for 2 weeks. The survival curve was generated using GraphPad Prism 8.0.1 software, and statistical significance was determined by the Log-rank test (**P＜0.01 and ****P＜0.0001). (**B**) Schematic phylogenetic tree of *Y. pestis* with *Y. pseudotuberculosis* at the root representing the ancestor. Branches 0–4 are shown in different colors with ancient DNA-related lineages colored brown. The “Big bang” node giving rise to branches 1–4 is indicated by a star and the *pla* I259T mutation is pointed out with a red arrowhead. Branches length does not represent the evolutionary time.

To evaluate the impact of Pla-mediated Ymt cleavage on *Y. pestis* virulence, BALB/c mice were subcutaneously (*s.c*.) infected with approximately 10 CFU of various *Y. pestis* strains. As depicted in Fig. 6A, mice infected with Δ*ymt::ymt*-K299A exhibited significantly extended survival compared to those infected with Δ*ymt::ymt*, indicating an enhanced virulence due to Pla-mediated Ymt cleavage, consistent with our previous findings (Fig. 5D). In addition, mice infected with Δ*pla::pla*-I259T, characterized by diminished Ymt cleavage efficiency, also displayed attenuated virulence compared to Δ*pla::pla* and no animal died when challenged with about 10 CFU of bacteria (Fig. 6A). According to the phylogenetic tree of *Y. pestis* based on the genome-wide single nucleotide polymorphisms (SNPs), the I259T substitution within *pla* has been acquired and maintained across all modern lineages of *Y. pestis* strains during its evolutionary trajectory (Fig. 6B). Our results suggest that the I259T substitution within Pla attenuates the virulence of *Y. pestis* in mice by reducing its cleavage activity toward Ymt, which prolongs the survival of infected animals and potentially widen the transmission window to new hosts.

## DISCUSSION

This study unravels a complex mechanism driving the evolution of *Y. pestis*, the intricate interplay between Ymt and Pla. These pivotal elements are encoded on the pMT1 and pPCP1 plasmids that are acquired during the evolution from ancestral *Y. pseudotuberculosis*. Ymt plays a crucial role in flea-bite transmission, while Pla is essential for dissemination within the host body following subcutaneous infection. Consequently, the interplay between these factors is not only particularly interesting but also pivotal for understanding the physiology and pathogenesis of *Y. pestis*.

Traditionally, Ymt was perceived as a cytoplasmic protein (17). However, Ymt’s substantial abundance and cleavage products in culture supernatants of various *Y. pestis* strains demonstrate that it is an extracellular protein capable of being secreted from the bacteria. One potential mechanism could be secretion *via* outer membrane vesicles shed from the bacteria during growth; however, we did not further explore the specific mechanisms involved in Ymt secretion. In this study, we identified Ymt as a novel substrate of Pla protease with the cleavage site pinpointed between amino acids K299 and V300 as determined by N-terminal sequencing. Experimental validation using Ymt_K299A_-expressing *Y. pestis* and the purified Ymt_K299A_ confirms that the K299A substitution of Ymt confers resistance to Pla cleavage, emphasizing the specificity of Pla in cleaving Ymt.

Pla has been found to cleave various substrates in mammalian hosts to facilitate the pathogenesis of *Y. pestis*. It also cleaves *Y. pestis* proteins, such as Yops secreted by Type III secretion system and chromosomally encoded protein KatY (13, 20). The majority of the identified cleavage sites are found located between a Lys residue and other amino acids (21), while the number of cleavage sites varies among different substrates. For example, Pla cleaves FasL at multiple sites to eventually degrade it and cleaves Prdx6 at three sites to generate four distinct peptides (22, 23). The identified cleavage site on Ymt highlights a unique single-site cleavage, resulting in the generation of two fragments.

Obviously, Pla has undergone a positive selection process, since a single amino acid substitution (I259T) in Pla was acquired and subsequently stabilized during the evolution of *Y. pestis*. This substitution in Pla appears to have conferred a selective advantage to the bacterium, resulting in its fixation within the *Y. pestis* population. Independent studies have illustrated that I259T substitution in Pla enhances the invasiveness of *Y. pestis* by improving its cleavage activity toward plasminogen (12, 14). Interestingly, we found that Pla-I259T exhibits a 50% reduction in cleavage rate toward Ymt compared to Pla. Protein structure prediction results revealed that the hydrophilic side chain of threonine (T) may enhance the binding of Pla to the substrate Ymt, thereby reducing the cleavage efficiency. One major finding of this study is that Ymt promotes the survival of *Y. pestis* in mouse blood, both *in vivo* and *in vitro*, with its PLD activity playing a crucial role in this process. These findings align with the crucial role of Ymt’s PLD activity in the survival of *Y. pestis* within the flea midguts, which are rich in blood ingested from hosts. In addition, the survival ability of *Y. pestis* in the blood is correlated with the bacterial load in the host’s blood, potentially influencing the efficiency of flea-borne transmission. The enhanced survival of Δ*ymt::ymt*-K299A compared to Δ*ymt::ymt* in the blood (Fig. 5C) clearly indicates that the Pla-mediated cleavage of Ymt could have a detrimental effect on bacterial survival within the host’s blood, potentially leading to a decreased level of bacteremia. Notably, the I259T substitution of Pla mitigates this effect by reducing its cleavage activity toward Ymt, thereby benefiting bacterial survival and resulting in higher bacteremia in the infected host. Furthermore, the I259T substitution prolongs the survival period of the infected mice (Fig. 6A), implying that it could benefit the transmission of *Y. pestis* to the next host. Consequently, our study reveals biological implications of Pla-mediated cleavage of Ymt, providing new insights into the evolutionary significance of I259T substitution in Pla.

The PLD activity of Ymt plays a vital role in the survival of *Y. pestis* within the flea midgut, and its absence leads to the incapacity of *Y. pestis* to form a blockage in the digestive tract of fleas (6). Our results showed that the *in vitro* biofilm formation capacity of *Y. pestis* was not reliant on the PLD activity of Ymt. However, a recent study has pointed out that the PLD activity of Ymt promoted the rapid formation of aggregation which was further developed to cause obstruction in fleas (24). This discrepancy suggested that the *in vitro* biofilm-forming capability of *Y. pestis* may not accurately represent its capability in forming a blockage in the flea guts, a crucial factor directly related to the transmissibility of strains. This observation also aligns with a previous study that identified *gmhA* as a gene required for flea blockage using a *Caenorhabditis elegans* biofilm system, whereas a *gmhA* mutant exhibited no discernible impact on *in vitro* biofilm formation assays (25). Together, we concluded that it should be cautious to conclude about *in vivo* biofilm formation capabilities of *Y. pestis* based on *in vitro* data. In addition, we found that Pla-mediated cleavage of Ymt reduces the production of biofilm *in vitro*. Thus, it would be valuable to compare the survival of Δ*ymt::ymt* and Δ*ymt::ymt*-K299A directly in fleas, as well as their efficacy in inducing flea blockage, which will further illustrate the roles of Pla-mediated cleavage of Ymt in the flea-borne transmission.

It is important to note that further exploration is needed to understand how Pla-mediated cleavage of Ymt influences biofilm formation and the survival of *Y. pestis* in the blood. However, we can infer that the two cleaved fragments of Ymt produced by Pla-mediated cleavage are separated *in vivo*, leading to the loss of the PLD activity and decreased survival of Δ*ymt::ymt* compared to Δ*ymt::ymt*-K299A in the blood. In addition, the two distinct fragments of Ymt, when separated *in vivo*, may potentially interact with other unidentified proteins that play critical roles in the biofilm formation of *Y. pestis*. Therefore, we believe that identifying proteins functionally influenced by Ymt or its cleaved fragments would contribute to a better understanding of the impact of Pla-mediated cleavage of Ymt on the phenotypes of *Y. pestis*. Virulence and transmissibility are two intricately balanced and essential metrics of pathogens. High virulence always leads to the rapid death of infected hosts, decreasing opportunities for pathogens to be transmitted to new hosts. To improve transmissibility, pathogens often evolve in the direction of virulence attenuation. Our results demonstrate that the Pla-mediated cleavage of Ymt enhances the virulence of *Y. pestis*, while strains expressing Pla-I259T characterized by reduced cleavage activity toward Ymt, exhibit decreased virulence than those expressing Pla. Taken together, this study elucidates how the Pla-mediated cleavage of Ymt modulates the virulence dynamics of *Y. pestis*, with Pla-I259T mutation emerging as a natural adaption that reduces virulence to animals, prolonging their survival and potentially enhancing transmissibility.

## MATERIALS AND METHODS

### Bacterial strains, cells, and culture conditions

The bacterial strains and plasmids used in this study are listed in Table S1. *Y. pestis* 201 strain belongs to the biovar Microtus and is highly virulent to mice but avirulent to humans. *Y. pestis* strains were cultured in Luria-Bertani (LB) broth at 26°C and *E. coli* DH5α or BL21(DE3) were grown in LB broth at 37°C. When necessary, antibiotics were added to the growth medium at the following concentrations: 100 µg/mL ampicillin, 34 µg/mL chloramphenicol, and 50 µg/mL kanamycin.

### Construction of the *Y. pestis* mutants and the complemented strains

The strains Δ*ymt* and Δ*pla* were constructed *via* homologous recombination using a CRISPR-Cas12a-assisted recombineering system (26). Briefly, two oligonucleotides consisting of 25-nt targeting regions of deletion genes with *Bsa*I cohesive ends were chemically synthesized and annealed before being cloned into pYC1000-eforRED-SacB by Golden Gate assembly. To generate dsDNA homologous arms for the target genes, 500 bp homologous sequences flanking the target genes at both sides of DNA fragments were amplified by PCR from genomic DNA of *Y. pestis* strain 201, followed by fusion-PCR. The gel-purified PCR products and pYC1000-eforRED-SacB containing the corresponding sgRNA were then introduced into *Y. pestis* strain 201 containing pKD46-FnCpf1 plasmid by electroporation. Bacteria were plated on LB agar plates containing ampicillin and chloramphenicol and the positive clones were verified by PCR amplification and sequencing. Two helper plasmids were then cured by incubating the cells in LB broth supplemented with 7% sucrose at 42°C.

To construct complemented strains, DNA fragments generated by PCR containing 300 bp-upstream sequence to the interested gene and the coding sequences of interested genes were ligated into plasmid pACYC184. The recombinant pACYC184 plasmids were then individually introduced into the corresponding mutants, yielding the complemented strains, which were confirmed by PCR and Western blotting (Ymt antibody and Pla antibody).

### Purification of recombinant proteins

*E. coli* BL21 (DE3) strains containing plasmids pET28a-Ymt, pET28a-Ymt-K299A, pET28a-Ymt-Fn, pET28a-Ymt-Fc, and pET28a-Ymt-H188*N* + H525N were grown in LB broth with 50 µg/mL kanamycin at 37°C in an incubator at 200 rpm. When OD_600nm_ of the cultures reached 0.6, 0.2 mM Isopropyl β-D-1-thiogalactopyranoside (IPTG) was added to induce protein expressions, and the cultures were transferred to grow overnight at 18°C in an incubator at 150 rpm. Proteins were then purified by nickel-nitrilotriacetic acid-agarose (Ni-NTA) affinity chromatography and desalted using PD-10 desalting columns (GE Healthcare, Pittsburgh, PA, USA). The concentrations of proteins were determined with a BCA protein assay kit (23227, Thermo Fisher Scientific, Rockford, USA) according to the manufacturer’s instructions and preserved at −80°C.

### Phospholipase D assays

Phospholipase D (PLD) assays were performed using the Phospholipase D activity assay kit (MAK137, Sigma-Aldrich, Saint Louis, MO. USA). Briefly, purified proteins Ymt, Ymt_K299A_, Ymt_H188N+H252N_, Ymt-Fn, and Ymt-Fc were diluted in assay buffer to the same molar concentration. 10 µL of each sample was mixed with 90 µL of the Master Reaction Mix and fluorescence was measured with a fluorescence microplate reader (λex = 530 nm; λem = 585 nm) according to the manufacturer’s protocols. Results are shown as means ± standard deviation. Data are representative of three independent experiments.

### Cleavage assays *in vitro*

*E. coli* K12 containing Pla-expressing plasmid or empty vector were cultured in LB broth to exponential phase before being collected and resuspended with equal amounts of Ymt or Ymt_K299A_ according to the results of BCA protein assay, respectively. After incubation at 37°C for 2 h, bacteria were removed by centrifugation. Proteins contained within the supernatants were diluted to the same molar concentration for subsequent PLD activity measurement as described above and an aliquot of corresponding protein without incubation with *E. coli* K12 containing Pla-expressing plasmid was utilized as positive control.

### Gel electrophoresis and western blotting analysis

After *in vitro* cleavage assays, proteins collected were boiled in SDS loading buffer for 10 min, separated by 12% SDS-PAGE, or mixed with native sample loading buffer and separated by 12% non-denaturing PAGE. Proteins on the SDS-polyacrylamide gel were subsequently transferred to polyvinylidene difluoride membranes and detected with anti-Ymt rabbit polyclonal antibody, followed by incubation with an IRDye 800CW-conjugated goat anti-rabbit secondary antibody. Images of the immunoblotting results were obtained using the Odyssey SA imaging system (LI-COR Biosciences, Lincoln, NE, USA).

### Cleavage assays *in vivo*

*Y. pestis* strain 201 and various mutants of *Y. pestis* were grown in 20 mL LB broth to OD_620nm_ 1.0 at 26°C and then 10 mL of the cultures were transferred to 37°C. All strains were then cultured for 5 more hours before being collected through centrifugation at 7,500 rpm for 10 min at 4°C. The bacterial pellets were weighed, resuspended in 5 mL PBS, mixed with an equal amount of 2 × SDS loading buffer, and boiled for 10 min. The supernatants were passed through a 0.22-µm filter and precipitated with 10% trichloroacetic acid (TCA) overnight. The resulting pellets were boiled in 2 × SDS loading buffer (100 µL/0.1 g bacterial pellets) for 10 min. Equal aliquots of samples from different bacterial strains were used for detection. Proteins in both supernatants and pellets of the cultures were separated by 12% SDS-PAGE and analyzed by immunoblotting with anti-Ymt and anti-Pla rabbit polyclonal antibodies.

### Assessment of the toxicity of protein in mice

Pla-expressing *E. coli* K12 was grown in LB broth at 37°C to OD_600nm_=1.0 before being collected, washed with PBS, and resuspended with PBS solutions containing 2 mg of Ymt or Ymt_K299A_, respectively. Aliquots of the purified Ymt and Ymt_K299A_ were used as controls. All samples were incubated at 37°C for 4 h and supernatants from each group were collected, respectively, by centrifugation. After passing through the 0.22 µm filters, the supernatants were subjected to PD-10 desalting columns (GE Healthcare, Pittsburgh, PA, USA) to remove any possible small molecules secreted by bacteria during incubation. The concentrations of protein in supernatants were then determined with a BCA protein assay kit (23227, Thermo Fisher Scientific, Rockford, USA) according to the manufacturer’s instructions. Protein in four groups were all adjusted to the concentration of 500 µg/mL and subjected to 5 serial fivefold dilutions with PBS. Five groups (*n* = 5) of female BALB/c mice (6–8 weeks) purchased from Vitalriver Company (Beijing, China) were intraperitoneally challenged with 100 µL of protein suspensions from different dilutions of each protein. The challenged mice were observed daily for 14 days and the survival curves for each group were plotted using GraphPad Prism (v.8.0.1, GraphPad Software, La Jolla, CA). The Median lethal doses (LD_50_) value of each group of protein in mice was calculated as previously reported (27).

### Colony morphology assays

Aliquots (5 µL) of glycerol stocks of various *Y. pestis* strains were spotted onto LB agar plates and incubated for 9 days at 26°C. Chloramphenicol was added to LB agar plates for all strains except for the wild-type and the *ymt* mutant strains. Images of morphologies of all colonies were acquired using a Canon 60D DSLR camera (Canon, Inc., Tokyo, Japan).

### Crystal violet staining assays

The bacterial biofilm formation ability was determined using the crystal violet staining method as described previously (28). Briefly, *Y. pestis* strains cultured in LB broth to OD_620nm_ 1.0 at 26°C were incubated for 8 h at 4°C for cold shock. The strains were then diluted 1:20 in LB broth and inoculated into 24-well tissue culture plates with 1 mL of culture in each well before being incubated at 26°C for 24 h. The culture medium containing the planktonic cells was removed from each well to determine the OD_620_ value. Wells with adherent biofilms were washed gently twice with 3 mL H_2_O and fixed at 80°C for 15 min. The adherent cells were then stained with 3 mL of 0.1% crystal violet for 15 min. After removing the solution, the wells were washed twice with 3 mL of H_2_O, and the bound dye was dissolved with 2 mL of dimethyl sulfoxide. The OD_570_ values were recorded, and the relative biofilm formation for each sample was indicated by calculating with the formula: 100×OD_570_/OD_620_.

### Assessment of bacterial survival in mouse blood *in vivo*

Different *Y. pestis* strains were cultured separately in LB medium at 26°C to OD_620nm_=1.0. Bacterial cells were collected by centrifugation followed by fivefold dilution with PBS. Female BALB/c mice purchased from Vitalriver Company (Beijing, China) were intravenously inoculated *via* the caudal vein with 100 µL of diluted bacterial suspension of each strain. After 4 h, mice were sacrificed and the blood was collected to determine the living bacterial number. The data were analyzed using GraphPad Prism (v.8.0.1, GraphPad Software, La Jolla, CA).

### Assessment of bacterial survival in mouse blood *in vitro*

Bacterial cells of *Y. pestis* strains Δ*ymt::ymt*-H188*N* + H525N cultured to OD_620nm_=1.0 were collected by centrifugation followed by 10-fold dilution with PBS. 20 µL of bacterial cells at an appropriate dilution gradient was mixed with 200 µL mouse blood collected by retro-orbital technique. Ymt was added into the mixture to reach a final concentration of 100 µg/mL while an aliquot of heat-inactivated Ymt was used as a negative control after incubation at 60°C for 40 minutes to inactivate the PLD activity. The living bacterial number in the blood was determined after cultivation at 37°C for 2 and 4 h.

### Assessment of the *Y. pestis* virulence in mice

*Y. pestis* strain 201, Δ*ymt::ymt*, Δ*ymt::ymt*-K299A, Δ*pla::pla,* and Δ*pla::pla*-I259T were grown in LB medium until OD_620nm_ of ~1.0. Bacterial cells were collected by centrifugation followed by 10-fold dilution with PBS. Groups (*n* = 10) of female BALB/c mice (6–8 weeks old) were challenged subcutaneously at the inguinal region with 100 µL of bacteria suspension at an appropriate dilution gradient (about 10 CFU of each strain). Chloramphenicol at 17 mg/kg or tetracycline at 10 mg/kg was injected intraperitoneally twice daily into mice infected with corresponding complemented strains to maintain the complemented plasmids. Mice were observed once daily continuously for 2 weeks and the survival curves for each group were plotted using GraphPad Prism (v.8.0.1, GraphPad Software, La Jolla, CA).

### Prediction of protein structure

The tertiary structures of protein were predicted using AlphaFold/2.3.1 software and visualized by PyMOL2.3.0 software.

### Molecular dynamics simulation

A simulation system of a ligand-protein complex is constructed in a cubic box (30 × 30 × 30 nm) using Packmol (v20.14.3). The topology of the ligand is generated by AMBER-GAFF (vGAFF2) and Gaussian 09. Protein topology is built using Amber99SB ILDN. The system is neutralized with CL^−^ ions and energy is minimized. NVT and NPT ensemble simulations are performed. All bonds are constrained using the LINCS algorithm. Simulations are run on a server (CPU: Xeon W-3275M, GPU: Tesla A100). Simulation data are recorded every 10 ps.

## SUPPLEMENTAL MATERIAL

Supplemental material mbio.01075-24-s0001.pdf Fig. S1-S4; Table S1.

ASM does not own the copyrights to Supplemental Material that may be linked to, or accessed through, an article. The authors have granted ASM a non-exclusive, world-wide license to publish the Supplemental Material files. Please contact the corresponding author directly for reuse.
