# Supplementary material for: Unveiling the dance of evolution: Pla-mediated cleavage of Ymt modulates the virulence dynamics of Yersinia pestis
Source: mBio. 2024 Jul 3;15(8):e01075-24. doi: 10.1128/mbio.01075-24 (PMC11323527; doi:10.1128/mbio.01075-24)
Supplement: Supplemental material — Fig. S1-S4; Table S1. [file mbio.01075-24-s0001.pdf]

Supplementary Figures

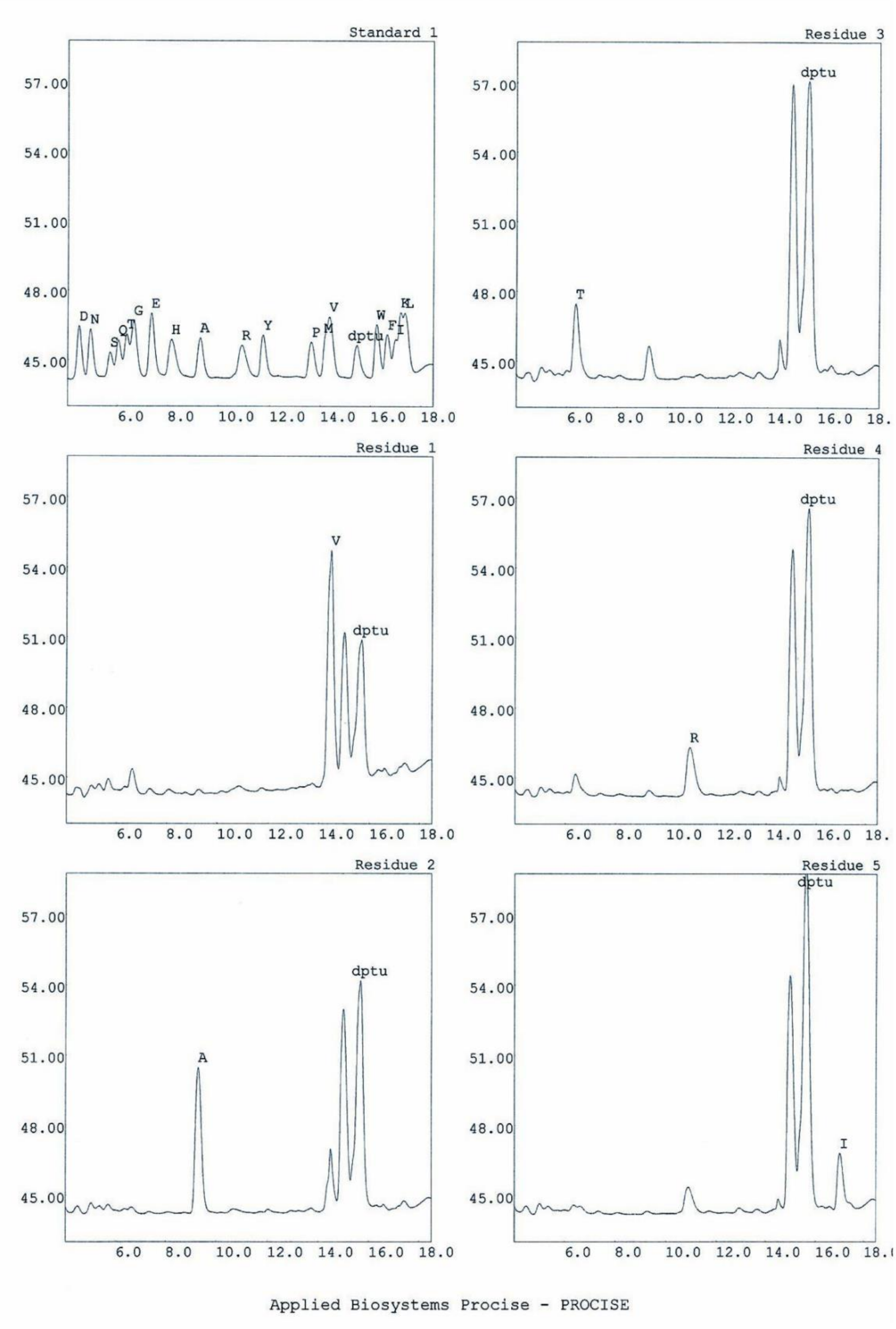

Fig S1. N-terminal sequence analysis of Ymt cleavage fragments. Residues VATRI are the N-terminal sequence of the cleavage fragment corresponding to the C-terminus of Ymt.

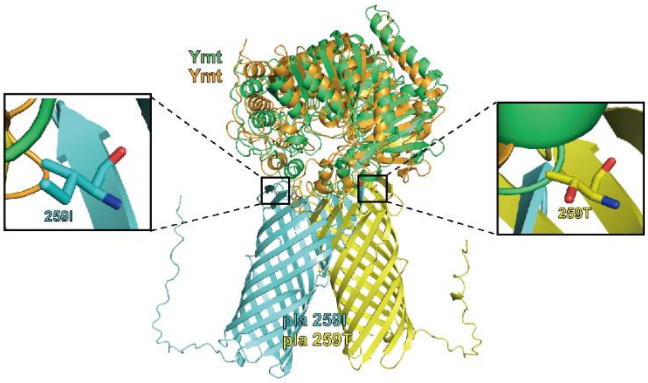

Fig S2. Prediction of the tertiary structure of Ymt-Pla and Ymt-Pla-I259T complexes by Alphafold2. The binding site between Ymt and Pla changed when a I259T mutation occurred within Pla.

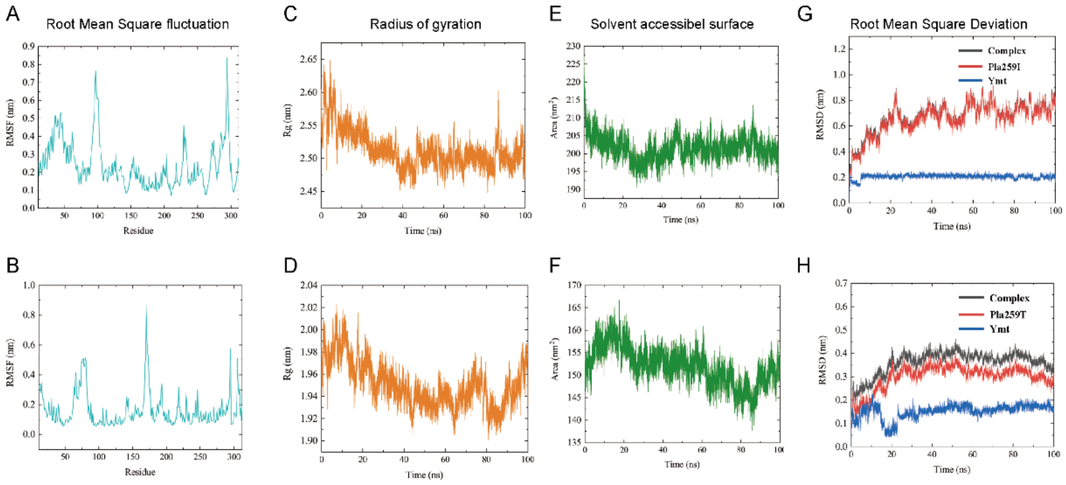

Fig S3. The molecular dynamic simulation of Ymt with different Pla variants. (A) The peaks of the root mean square fluctuation (RMSF) curve of the Ymt-Pla and Ymt-Pla-I259T (B) complexes mainly located in the LOOP region of the protein, indicating that the fluctuation of the LOOP region is large. (C) The radius of gyration value of the Ymt-Pla and Ymt-Pla-I259T (D) complexes shows a downward trend, indicating that the protein structure becomes more compact during the simulation process. (E) The solvent accessible surface value of the Ymt-Pla and Ymt-Pla-I259T. (F) Complexes decreases with increasing simulation time, indicating that the area of the complex exposed to the solvent decreases. (G) The RMSD for Ymt-Pla and Ymt-Pla-I259T. (H)

Complexes tended to stabilize at the average values of 0.7291 and 0.3578 when the simulation time reached 40 ns.

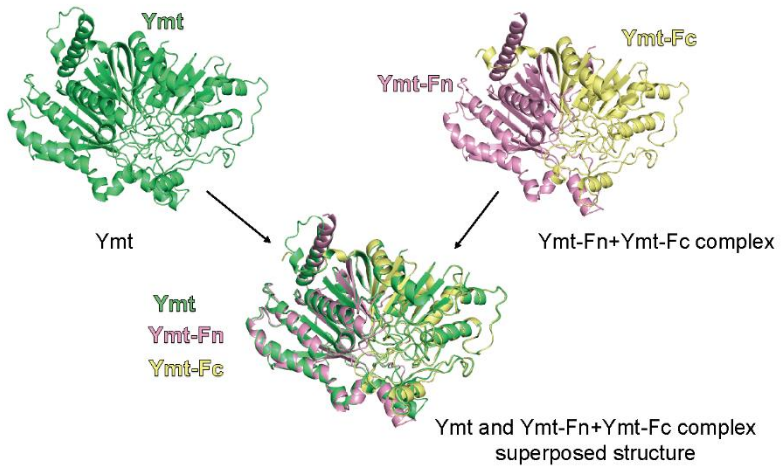

Fig S4. Prediction of the tertiary structure of Ymt and Ymt-Fn+Ymt-Fc complex by Alphafold2. The conformation of the Ymt-Fn+Ymt-Fc complex closely resembled that of the intact Ymt monomer.

Supplementary Tables

Supplementary Table 1. Strains and plasmids used in this study

**Strains or plasmid**	**descriptions**	**sources**
**Strains**		
Y. pestis strains		
201	Wild-type *Y. pestis* biovar Microtus strain	(1)
KIM D27	*Y. pestis* biovar Medievalis strain with a deletion of chromosomal pigmentation locus	lab stock
Δ*ymt*	201 with deletion of *ymt* using CRISPR-Cas12a-assisted recombineering system	this study
Δ*pla*	201 with deletion of *pla* using CRISPR-Cas12a-assisted recombineering system	this study
Δ*ymt*::*ymt*	Δ*ymt* carrying plasmid pACYC184-*ymt* (Cm^r)	this study
Δ*ymt*::*ymt*-K299A	Δ*ymt* carrying plasmid pACYC184-*ymt*-K299A (Cm^r)	this study
Δ*ymt*::*ymt*-Fn	Δ*ymt* carrying plasmid pACYC184-*ymt*-Fn (Cm^r)	this study
Δ*ymt*::*ymt*-Fc	Δ*ymt* carrying plasmid pACYC184-*ymt*-Fc (Cm^r)	this study
Δ*ymt*::*ymt*-H188N	Δ*ymt* carrying plasmid pACYC184-*ymt*-H188N (Cm^r)	this study
Δ*ymt*::*ymt*-H525N	Δ*ymt* carrying plasmid pACYC184-*ymt*-H525N (Cm^r)	this study
Δ*ymt*::*ymt*-H188N+H525N	Δ*ymt* carrying plasmid pACYC184-*ymt*-H188N+H525N (Cm^r)	this study
Δ*pla*::*pla*	Δ*pla* carrying plasmid pACYC184-*pla* (Cm^r)	this study
Δ*pla*::*pla*-I259T	Δ*pla* carrying plasmid pACYC184-*pla*-I259T (Tet^r)	this study

E. coli strains		
K12	a strain of *E. coli*	lab stock
K12-vecor	K12 carrying plasmid pACYC184	this study
K12-Pla	K12 carrying plasmid pACYC184-*pla* (Cm^r)	this study
K12-Pla-I259T	K12 carrying plasmid pACYC184-*pla*-I259T (Cm^r)	this study
BL21(DE3)	strain used for recombinant proteins purification	lab stock
DH5α	strain used for stocking the constructed plasmids	lab stock
**Plasmids**		
pKD46	Temperature-sensitive plasmid expressing λ-Red recombinase under the control of arabinose, Ampr	lab stock
pKD46-FnCpf1	pKD46 inserted with the FnCpf1 open reading frame (ORF) sequence, Ampr	(2)
pYC1000-eforRED-SacB	Sucrose-sensitive plasmid used for inserting 25-bp targeting sequence for specific genes, Cmr	this study
pACYC184-*ymt*	sequence of *ymt* gene was inserted into pACYC184, Cmr	this study
pACYC184-*ymt*-K299A	sequence of *ymt* gene with a point mutation in K299 was inserted into pACYC184, Cmr	this study
pACYC184-*ymt*-Fn	amino acid 1-299 coding sequence of *ymt* gene was inserted into pACYC184, Cmr	this study
pACYC184-*ymt*-Fc	amino acid 300-587 coding sequence of *ymt* gene was inserted into pACYC184, Cmr	this study

pACYC184-*ymt*-H188N	sequence of *ymt* gene with a point mutation in H188 was inserted into pACYC184, Cmr	this study
pACYC184-*ymt*-H525N	sequence of *ymt* gene with a point mutation in H525 was inserted into pACYC184, Cmr	this study
pACYC184-*ymt*-H188N+H525N	sequence of *ymt* gene with point mutations in H188 and H525 was inserted into pACYC184, Cmr	this study
pACYC184-*pla*	sequence of *pla* gene was inserted into pACYC184, Cmr	this study
pACYC184-*pla*-I259T	sequence of *pla* gene with a point mutation in I259 was inserted into pACYC184, Tetr	this study
pET28a-Ymt	plasmid for purification of Ymt, Kan^r	this study
pET28a-Ymt-K299A	plasmid for purification of Ymt-K299A, Kan^r	this study
pET28a-Ymt-Fn	plasmid for purification of Ymt-Fn, Kan^r	this study
pET28a-Ymt-Fc	plasmid for purification of Ymt-Fc, Kan^r	this study
pET28a-Ymt-H188N+H525N	plasmid for purification of Ymt-H188N+H525N, Kan^r	this study

1. Y. Song, Z. Tong, J. Wang, L. Wang, Z. Guo, Y. Han, J. Zhang, D. Pei, D. Zhou, H. Qin, X. Pang, Y. Han, J. Zhai, M. Li, B. Cui, Z. Qi, L. Jin, R. Dai, F. Chen, S. Li, C. Ye, Z. Du, W. Lin, J. Wang, J. Yu, H. Yang, J. Wang, P. Huang, R. Yang, 2004. Complete genome sequence of Yersinia pestis strain 91001, an isolate avirulent to humans. DNA Res http://dx.doi.org/10.1093/dnares/11.3.179

2. M. Y. Yan, H. Q. Yan, G. X. Ren, J. P. Zhao, X. P. Guo, Y. C. Sun, 2017.

CRISPR-Cas12a-Assisted Recombineering in Bacteria. Appl Environ Microbiol http://dx.doi.org/10.1128/AEM.00947-17
